# Label-Free Proteomics of Severe Acute Hepatitis of Unknown Origin in Children by High-Resolution Mass Spectrometry

**DOI:** 10.1021/acsomega.4c08745

**Published:** 2024-12-13

**Authors:** Josivan Barbosa de Farias, Maria Luiza de Lima Vitorino, Fabrício Andrade Martins Esteves, Eduardo Jorge da Fonseca Lima, Roberto Afonso da Silva, José Luiz de Lima Filho

**Affiliations:** † Universidade Federal de Pernambuco—Instituto Keizo Asami iLIKA. Av. Prof. Moraes Rego, 1235-Cidade Universitária, 50670-901 Recife, Pernambuco, Brazil; ‡ Centro Universitário Tabosa de Almeida. Av. Portugal, 584-Bairro Universitário, 55016-400 Caruaru, Pernambuco, Brazil; § IMIP Hospital—Instituto de Medicina Integral Professor Fernando Figueira. Rua dos Coelhos, 300-Boa Vista, 50070-902 Recife, Pernambuco, Brazil

## Abstract

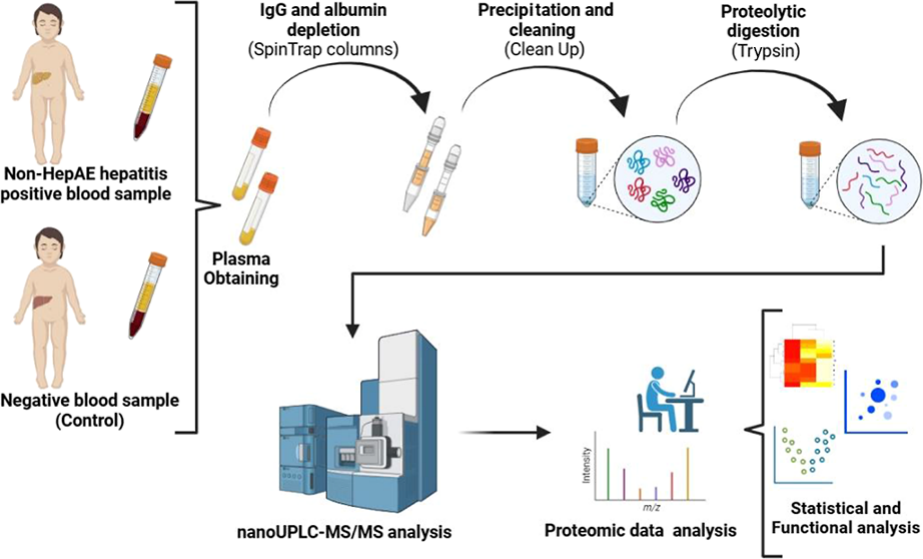

Acute hepatitis of unknown etiology (non-HepA-E hepatitis) emerged affecting children in 2021 and in parallel with the COVID-19 pandemic. In the present article, we performed an analysis between two plasma samples from pediatric patients, one with non-HepA-E hepatitis and the other healthy, to evaluate possible proteomic alterations associated with viral targets as possible causative agents and pathophysiological processes using the high-resolution and label-free LC–MS/MS technique. We identified 72 altered differentially expressed proteins, 45 upregulated and 27 downregulated. Gremlin-1, a protein associated with tissue fibrosis, was detected exclusively in the positive sample. Proteins involved in immunological processes, coagulation cascade, complement cascade, lipid transport, oxidative stress, acute inflammatory response, and those related to extracellular matrix deposition were also identified. In addition, some proteins of viral origin were detected, mainly from respiratory viruses. Proteomic studies of diseases such as hepatitis and other hepatopathologies have become essential for understanding pathophysiological processes and detecting molecular triggers.

## 1 Introduction

In April 2022, the World Health Organization (WHO) warned about cases of severe hepatitis in some children in the United Kingdom. Most of the identified cases intensified in Europe and the world scenario; cases were detected in several countries in Europe, United States, and Asia.^1,2^ It was a hepatitis with a high degree of severity specifically affecting children and of unknown etiology that differs it from other existing hepatitis.^3,4^

In diagnosing these cases, routine tests to assess liver damage, such as the transaminase index, demonstrated positive results, but immunoserology for viral hepatitis (hepatitis A, B, C, D, and E) and autoantibodies for autoimmune hepatitis were negative in all patients evaluated, characterizing it as hepatitis of unknown origin, non-HepA-E hepatitis.^5^

The appearance of cases of non-HepA-E hepatitis in children generates a negative impact on the health system, given the difficulty in treatment due to the uncertainty of a specific target, as occurs in other hepatitis already known, as well as the rapid worsening of the disease often generates severe liver damage such as fibrosis and thus increases the chances of needing a transplant.^6,7^

It is hypothesized that an immune-mediated response in the liver may be the key to understanding the main etiology where non-HepA-E hepatitis is established.^8^ Some of the cases reported in the scientific literature demonstrated previous infection with adenovirus,^9^ in addition to other viruses such as adeno-associated virus-2 (AAV-2), human herpesvirus-6 (HHV-6),^10,11^ syncytial virus respiratory, and influenza A virus,^12^ and other patients had a history of previous SARS-CoV-2 infection.^13,14^

A postinfectious hepatic immune response is suggested, which could generate an exacerbated inflammatory process similar to that which occurs in Pediatric Multisystemic Inflammatory Syndrome (P-SIM), possibly caused by immune dysregulation and consequently the formation of T-cell activating superantigens.^15,16^

The study of proteins involved in the pathophysiological processes of hepatitis of already known etiology (HepA-E) has proven to be of great importance, given the versatility that these biomolecules have in acting in important biochemical pathways such as in immune processes, replication of pathogenic agents, and cell signaling.^17−19^

Proteins can become important molecular therapeutic targets for choosing possible treatment strategies, in addition to establishing themselves as excellent biomarkers for a better clinical diagnosis.^20^ Mass spectrometry is a promising technique, especially in clinical proteomics, and has been promoting the identification of proteins related to the pathophysiology of hepatitis, as well as those promising as possible biomarkers and potential inhibitors of disease progression.^21^

As it is a rare and recently identified pathology, the few cases compared to other hepatitis make it difficult to obtain a greater number of samples of non-HepA-E hepatitis. Given these factors, the objective of this article was to study a clinical case of this disease, seeking to analyze, through label-free proteomics, proteins possibly involved in pathophysiological processes as well as elucidate associated factors that induce the infectious process and the progression of liver damage.

## 2 Results

### 2.1 Proteomic Data Analysis and Statistical Analysis

In total, 206 proteins were identified, of which 72 were differentially expressed proteins (DEPs). From the identification of DEPS, 45 proteins were upregulated and 27 downregulated in the positive sample for hepatitis non-HepA-E. Furthermore, the Gremlin-1 (GREM1) protein was identified as exclusive in the positive sample (max fold charge infinity). Among the upregulated DEPs were immune system proteins such as immunoglobulin gamma, alpha, delta, heavy, and various light chain variables Kappa and Lambda, respectively in addition to other proteins such as TRAF3-interacting protein 1, galectin-3-binding protein, and carboxypeptidase N.

Viral proteins were also detected, which were mostly related to viral replication processes. Within the upregulated proteins, we detected replication protein E1 from human papillomavirus 30 and nonstructural polyprotein 1A from human astrovirus-1. And with downregulation, we detected RNA-directed RNA polymerase L from the Seoul virus (strain 80–39), RNA-directed RNA polymerase L from human respiratory syncytial virus B (strain B1), hemagglutinin from the influenza A virus (strain A/Japan/305/1957 H2N2), and the replicase polyprotein 1ab from human coronavirus NL63.

The identified downregulated proteins include complement cascade proteins (complement C1r subcomponent, C3, C4-A, C4-B, C5, component C8 alpha chain, component C8 beta chain, factor I, and factor B), several apolipoproteins (APOA1, APOA2, APOA4, APOB-100, APOC2, APOC3, and L1), as well as other proteins that interact with apolipoproteins, such as hemopexin (HPX) and haptoglobin (HP). Some other proteins associated with the coagulation cascade were also identified as alpha-2-antiplasmin (SERPINF2), plasma serine protease inhibitor (SERPINA5), interalpha-trypsin inhibitor heavy chain H1 (ITIH1), interalpha-trypsin inhibitor heavy chain H4 (ITIH4), plasminogen (PLG), heparin cofactor 2 (SERPIND1), and coagulation factor X (F10).

**Figure 1:**
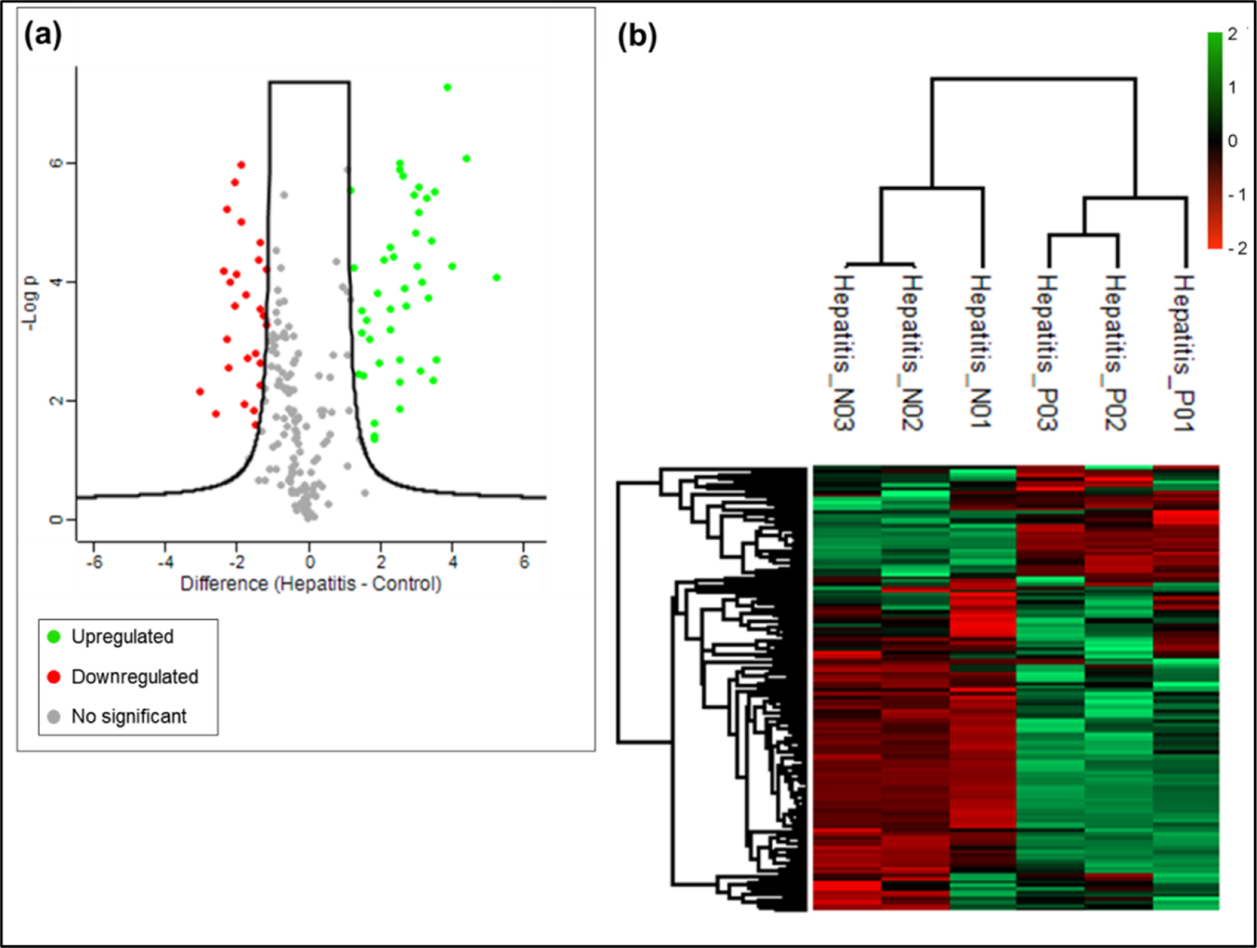
(a) Volcano plot. Distribution of DEPs with greater significance was compared between test and control samples. On the *x*-axis, we used log2 of Folder charge and on the *y*-axis −log10 *p*-value. (b) Heat map of DEPs, showing upregulated (*z*-score values: from 0 to 2) and downregulated (*z*-score values: from 0 to −2) proteins. Non-HepA-E hepatitis (positive) replicates: P01, P02, and P03. Control (negative) replicates: N01, N02, and N03.

Extracellular superoxide dismutase [Cu–Zn] (SOD3) was also downregulated, being an enzyme belonging to the antioxidant complex and important in fighting free radicals. Through the heat map (Figure 1b) obtained by analyses carried out by the PERSEUS software, we can observe that there was a greater amount of upregulated proteins compared to the standard.

After statistical analysis, the results showed which DEPs were significant when comparing the test and control samples. As we can see through the q value, many of the proteins obtained a value below 0.01 [according to the false discovery rate (FDR) value established at the time of analysis], which are considered significantly expressed. Among those of greatest importance in upregulation were proteins associated with the immune system, such as immunoglobulins, including gamma globulins, alpha immunoglobulins, and some with variable light chain subunits Kappa and Lambda, respectively (Figure 1a).

Of the most significantly upregulated viral proteins were replication protein E1 from human papillomavirus type 30, and no-structural polyprotein 1A from human astrovirus-1. The RNA-directed RNA polymerase L protein from human respiratory syncytial virus B (strain B1) was downregulated within the significance range.

Within the most significantly downregulated DEPs, several proteins of the complement cascade were identified, such as complement C5, factor B, component C8 alpha chain, C8 beta chain, factor I, and component C8 beta chain. Other proteins involved in oxidative stress such as extracellular superoxide dismutase [Cu–Zn] and some related to lipid transport, such as apolipoproteins A-I (APOA1), A-II (APOA2), A-IV (APOA4), and C–III (APOC3), were also significantly downregulated.

**Figure 2:**
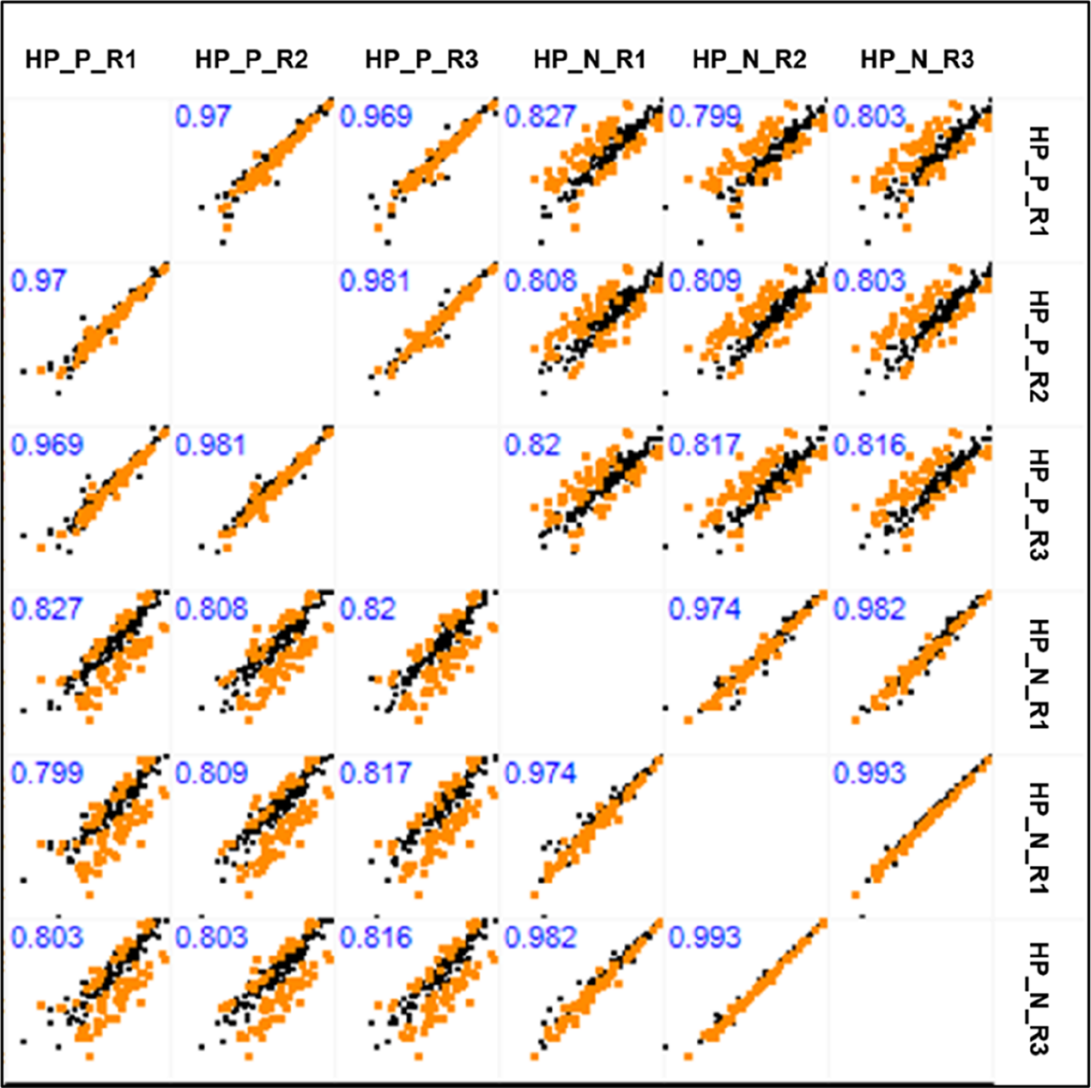
MultiScatter plot with Pearson’s correlation values between replicates of hepatitis non-HepA-E (positive) and control (hepatitis negative) test samples. Correlation values are highlighted in blue. Hepatitis non-HepA-E (positive) replicates: HP_P_R1, HP_P_R2, and HP_P_R3. Control replicates (negative): HP_N_R1, HP_N_R2, and HP_N_R3.

We can observe that the correlation between the replicates in each group, test, and control, respectively, were in the ideal margin, if we take into account Pearson’s correlation values. In Figure 2, we can observe that the correlation values between replicates of the same group were above 0.9 both in the test and control groups.

Through a multianalysis of the comparison between all replicates within each group, as well as comparisons made between replicates from different groups, we can observe that the Pearson correlation values between replicates from the same group (test × test/control × control) showed better value, all above 0.96. As analyses were carried out between replicates of different groups (test × control), the correlation values decreased, reaching 0.77 (Figure 2).

### 2.2 Protein–Protein Interaction and Functional Analysis

**Figure 3:**
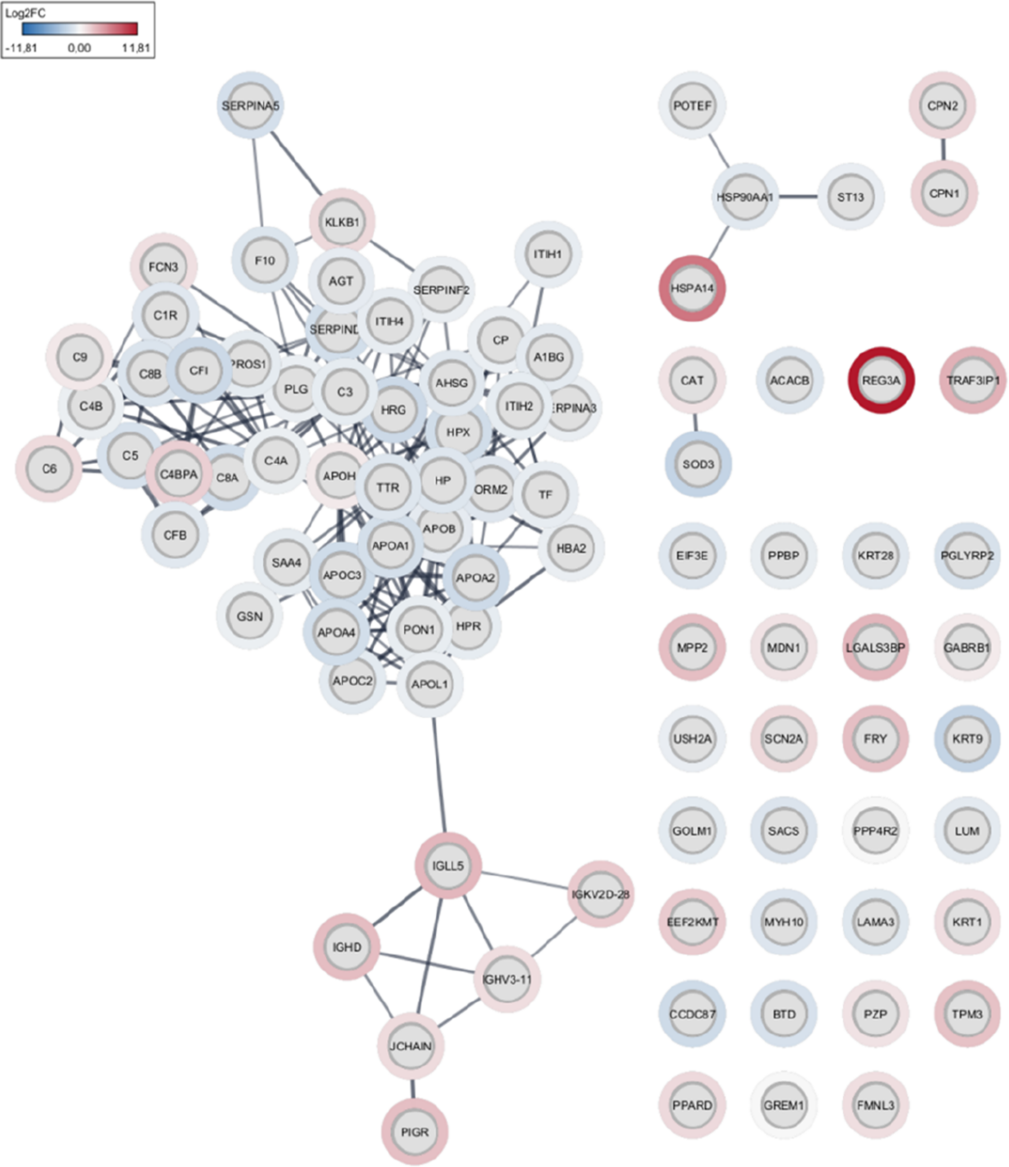
Protein–protein interactome obtained from Cytoscape. Log(Fold change) values show upregulated proteins in red and downregulated proteins in blue.

Alpha-2-antiplasmin, one of the DEPs with downregulation compared to the control sample, has direct interaction with other proteins that strongly regulate the coagulation cascade and are correlated with the formation of the extracellular matrix in fibrotic processes such as interalpha-trypsin inhibitor heavy chain H4 (ITIH4), inter- alpha-trypsin inhibitor heavy chain H1 (ITIH1), plasminogen (PL), heparin cofactor 2 (SERPIND1), plasma serine protease inhibitor (SERPINA5), alpha-2-HS-glycoprotein (AHSG), and the coagulation factor X (F10) (Figure 3).

**Figure 4:**
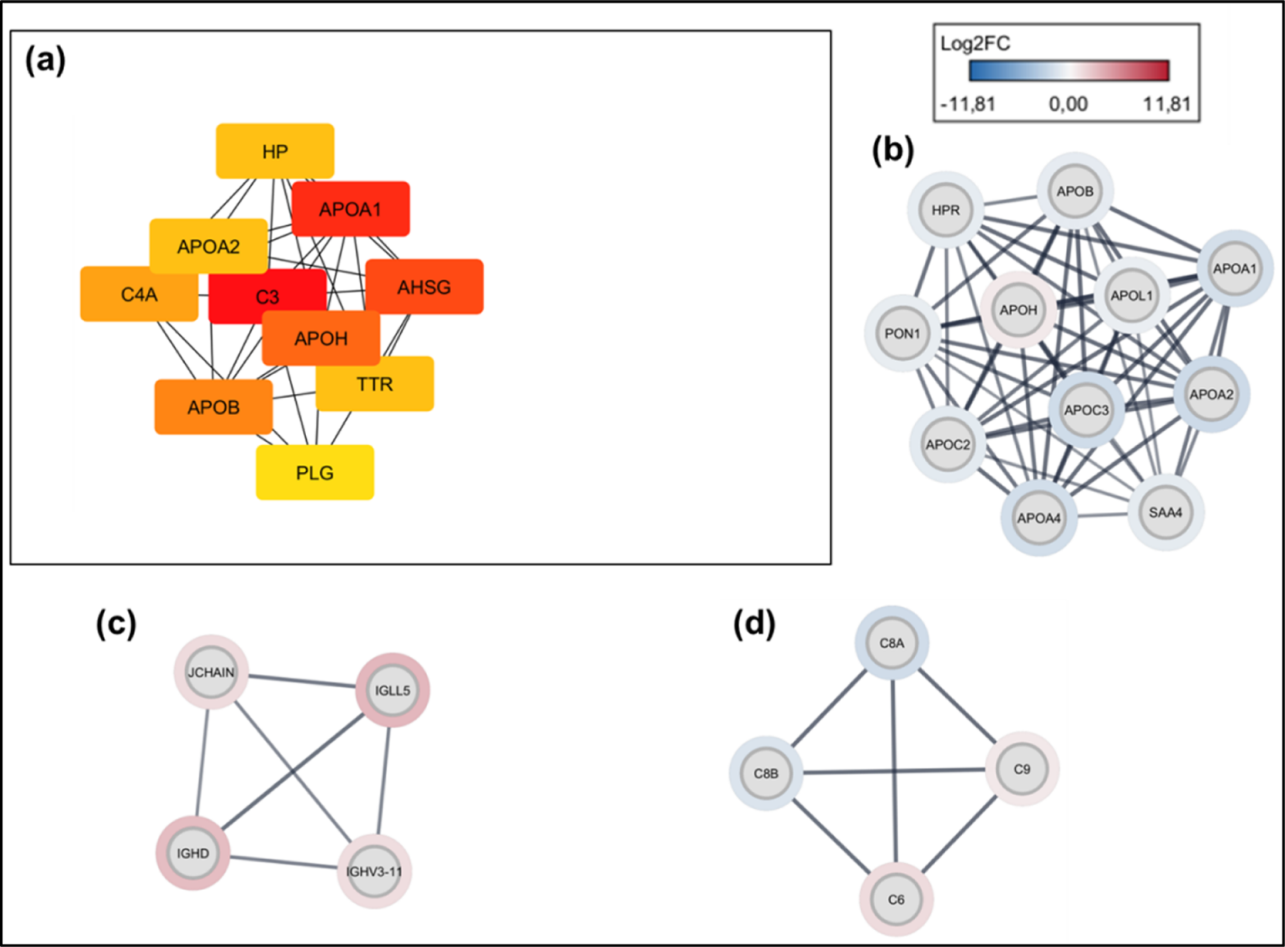
(a) Hub genes: those equivalent to the most connected proteins in the interaction network. (b) Cluster of interactions between apolipoproteins. (c) Cluster of interactions between immunoglobulins. (d) Cluster of interactions between proteins of the complement cascade.

We also observed that several apolipoproteins identified as downregulated interact with others of the same class but with upregulation, as can be seen by the difference between the Log Fold change values (Figure 4b). In addition, APOA1, APOA2, APOB, and APOH interact with haptoglobin (HP) and transthyretin (TTR) and plasminogen (PLG), the latter proteins being important in coagulation processes. We can observe this detail in the most connected genes in the interaction network (hub genes) according to the degree of interaction (Figure 4a).

Proteins associated with immune processes also showed clusters of strong interaction. Some of the immunoglobulins found upregulated were IGH5, IGHD, IGHV3-11, and JCHAIN (Figure 4c). And some were associated with the complement cascade: C8A, C8B, C6, and C9 (Figure 4d).

**Figure 5:**
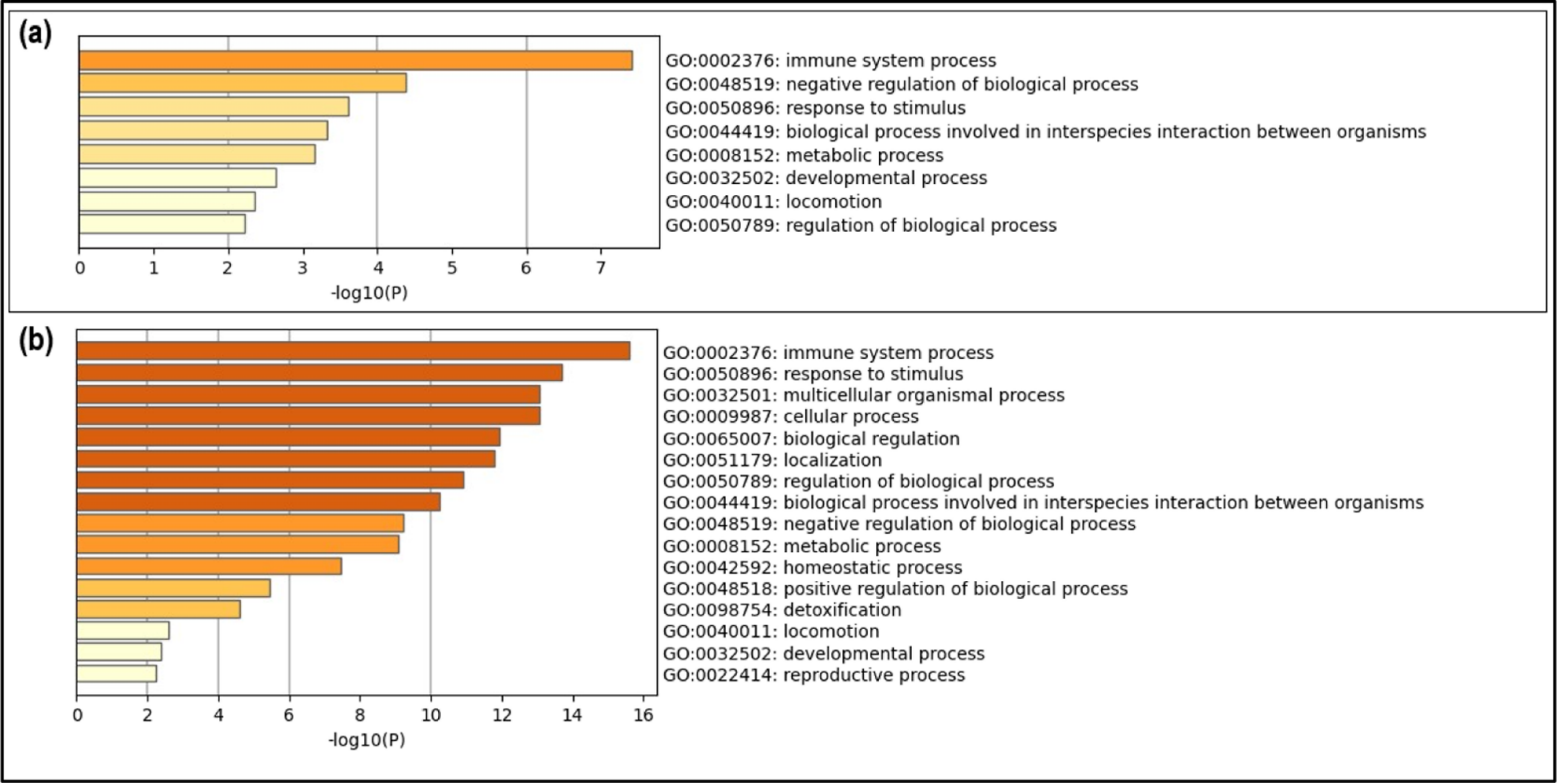
Enrichment analysis of gene expression and biological processes involved in the identified proteins. Upregulated protein enrichment analysis (a) and downregulated protein enrichment analysis (b).

In the functional analysis of pathways associated with biological processes, it was observed that most of the proteins analyzed act in pathways involved in immunological processes, which can consequently be correlated with the significant increase in the regulation of immunoglobulins, mainly due to the negative regulation of complement proteins in our MS/MS analyses (Figure 5).

In addition, other identified proteins have a direct interaction with proteins classified as important cytokines in immune response and inflammation mechanisms, such as the interaction of Gremlin-1 with TGF-β and TRAF3IP1 with TRAF3 and, consequently, with activators of inflammation pathways and T cells such as TNF-α and NFKB.

**Figure 6:**
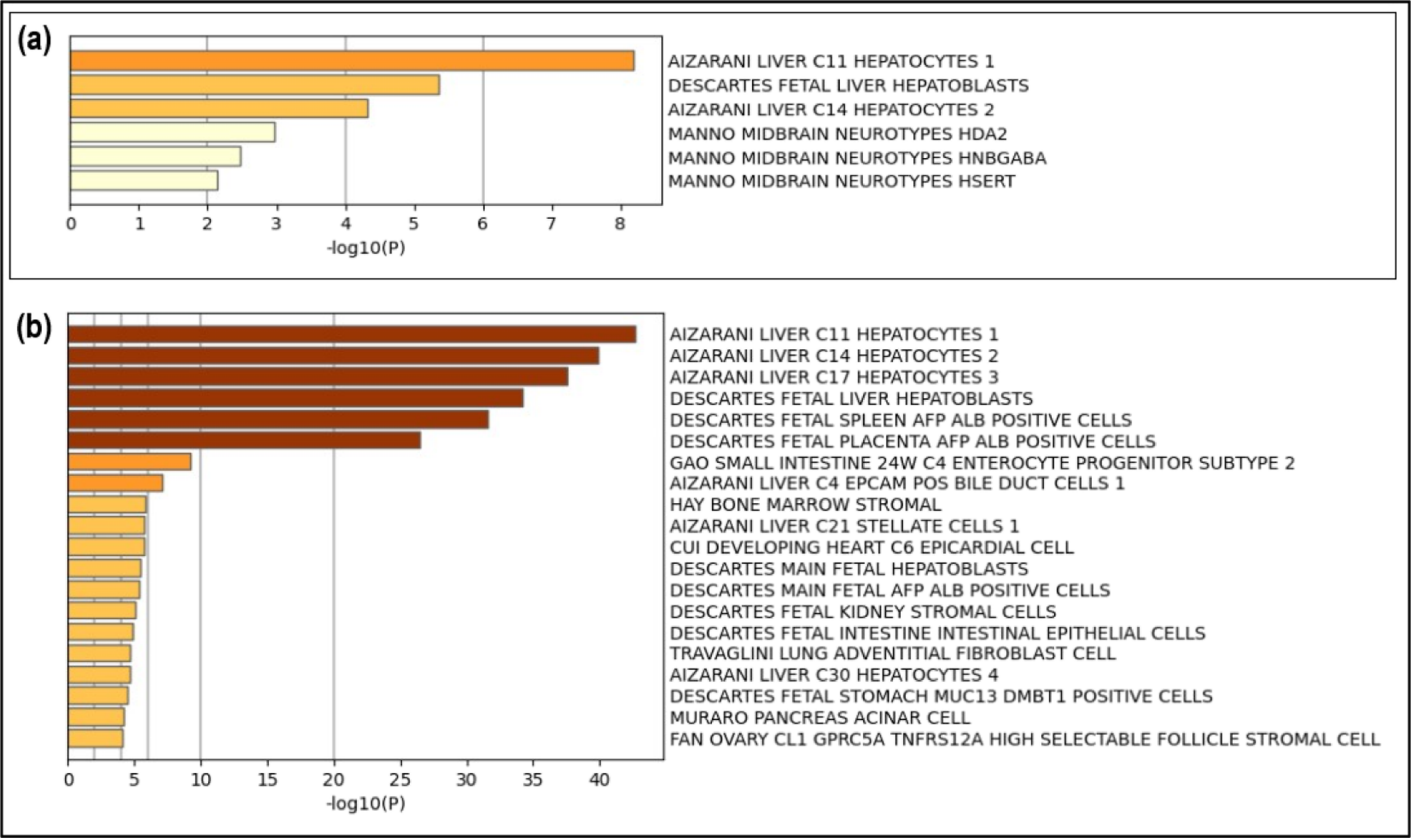
Analysis of the cell signature enrichment by gene expression. Relationship with C11, C14, and C17 hepatocyte lineages were the most prevalent. Upregulated protein functional analysis (a). Downregulated protein functional analysis (b).

With the evaluation of the cell signature by gene expression, it was possible to identify the highest specificity of up- and downregulated proteins with hepatocyte cell lines, with those of the C11, C14, and C17 lineage^22^ being those of higher prevalence (Figure 6).

**Figure 7:**
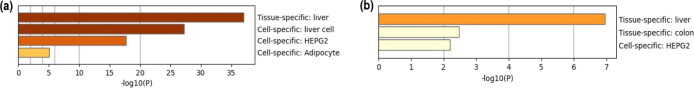
Functional analysis and specific tissue of protein action. The liver was identified as the target organ for the DEPs analyzed. Upregulated protein functional analysis in (a) and downregulated protein analysis in (b).

As a specific tissue, the liver was identified as the organ of action of the processes to which the identified proteins are associated, being strongly confirmed by the cellular signature of the hepatocytes, which demonstrates that the events that occurred in the pathology studied are correlated to the liver. (Figure 7).

## 3 Discussion

The Gremlin-1 protein (GREM1) was found only in the sample positive for non-HepA-E hepatitis in all replicates analyzed and with upregulation. The process of liver fibrosis is the chronic stage and the main aggravating factor of the most diverse types of hepatopathologies, including hepatitis. The upregulation of Gremlin-1 demonstrated its importance in the reduction of hepatoprotection as well as in the possible induction of liver fibrosis in some diseases that affect the liver due to its increased expression. Gremlin-1 promotes an increase in transforming growth factor-beta (TGF-β) which consequently can increase the activation of hepatic stellate cells causing fibrosis.^23,24^

Other proteins considered to block TGF-β are of great importance in hepatoprotection, as they reduce liver damage, as occurs in fibrosis resulting from hepatitis. Proteins such as BMP and activin membrane-bound inhibitor homologue (BAMBI), which with the inhibitory action of TGF-β, favor the signaling of important pathways that participate in the process of reducing fibrogenesis.^25,26^ Our results also detected upregulation of alpha 2 antiplasmin (SERPINF2). In the process of liver fibrogenesis, there is an increase in proteins such as fibronectin and collagen that can lead to the formation of complexes in the extracellular matrix (ECM) capable of altering the integrity of liver cells.^27,28^

Plasmin is characterized as an important serine protease responsible for the degradation of proteins present in the ECM structure, and its main inhibitor is SERPINF2.^29^ In fibrotic processes, there is a large increase in proteins in the ECM and consequently the need for plasmin action. Thus, negative regulation of SERPINF2 is capable of generating a significant increase in the proteolytic enzyme plasmin in its activated form. In the study by Chan et al., (2006)^30^ a decrease in the expression of SERPINF2 was detected in patients infected with HBV in the acute phase.

Another protein possibly associated with liver damage and identified with upregulation was TRA3-interacting protein 1 (TRAF3IP1) mainly through direct interaction with other important cytokine signaling proteins, such as TNF receptor-associated factor 3 (TRAF3). Hu et al. (2016)^31^ demonstrated that TRAF3 is a potential protein in promoting liver damage and increased inflammation via the mitogen-activated protein kinase 7 (MAP3K7)-dependent activation pathway and nuclear factor kappa B (NF-kB).

The regulation of important pathways related to NF-kB is defined in autoimmune diseases, as the increase in its signaling contributes to autoimmunity and chronic inflammatory processes by activating T cells.^32,33^ Thus, there is the possibility of TRAF3 in the direct activation of immune complexes by cytokine signaling, lymphocyte-mediated immune response, and consequent increase in hepatic hyperimmune activity.

The overexpression of immunoglobulins (Ig) present in the test sample (non-HepAE hepatitis) demonstrates the possible hepatic hyperimmune response similar to cases of autoimmune hepatitis.^34^ Because in addition to the significant increase in the expression of Ig, mainly alpha and gamma chain globulins (IgG), they can be an indication of the immune response in the liver due to the presence of possible infectious agents that promote the function of triggers.^35^

The downregulation of complement cascade proteins suggests a dysregulation of the immune system due to a deficiency in the elimination of immune complexes, which favors the process of immunopathogenesis.^36^ These factors automatically trigger an inability to eliminate immune complexes and cells that are important for the apoptosis process, thus being able to stimulate the synthesis of autoantibodies in the affected tissue. In addition, the effect of generating immune complexes brings with it the high possibility of generating autoantigens and consequently an increase in the hepatic immune-mediated response.^37^

Furthermore, given the possibility that pathogens of viral origin are correlated with the dissemination and severity of non-HepA-E hepatitis, we sought to verify which proteins could act as a trigger for a hepatic hyperimmune response but were unsuccessful in this study. We can highlight in the scientific literature the identification that some children diagnosed with hepatitis non-HepA-E had previous infection with Sars-CoV-2, in addition to others with the potential to activate an exacerbated immune response, such as adenovirus and other variants of SARS-CoV-2.^5,13,38^

We can take into account the possibility that biomolecules such as structural and nonstructural proteins of viruses, such as those found in this study, become superantigens capable of generating signaling in important cells of the immune system such as T cells, thus generating a hyperimmune reaction response in liver tissue initiating the acute phase of the disease.^15,39^ The possibility of preinfection by more than one different virus, such as adenovirus, adeno-associated virus, and SARS-CoV-2, could also be an important factor correlated with the formation of superantigens and, consequently, greater T-cell immunomediation and increased of liver damage.^12^

A galectin-3 binding protein (LGALS3BP) was also significantly increased in the non-HepAE acute hepatitis sample of the present study. LGALS3BP has been described in the scientific literature as being associated with liver diseases such as cirrhosis and NAFLD nonalcoholic fatty liver disease.^40^ It is considered a potential biomarker for NAFLD in addition to being related to the progression of liver disease causing cirrhosis and mainly fibrosis in the liver affected by HCV.^41,42^ Thus, the increase in LGALS3BP may be correlated with the rapid increase in severity characteristic of non-HepAE hepatitis, mainly due to the association of this protein with fibrogenesis in liver diseases.

Starting from proteins mainly associated with lipid transport and so important in cholesterol metabolism, we observed that many apolipoproteins identified were negatively regulated, as well as the negative regulation of haptoglobin and hemopexin, proteins strongly involved in the activity of apolipoproteins.^43^ The decrease in all of these proteins shows that a possible increase in cholesterol and triglycerides correlates with an increase in liver damage seen in high-severity hepatitis and nonalcoholic fatty liver disease.^44,45^

Patients with high fibrogenesis tend to have reduced expression of some apolipoproteins. It has been reported that APOC2 and APOC4 proteins are decreased in patients with advanced fibrosis due to nonalcoholic fatty liver disease—aggravated NAFLD.^46^ Other apolipoproteins such as APOB (apolipoprotein B-100) also correlate with liver disease in its aggravated stage. Proteins such as APOA2 and haptoglobin, in addition to having interaction pathways with each other, are also associated with acute inflammatory responses, being one of the main causes that favor the progression of hepatopathologies.

Extracellular superoxide dismutase [Cu–Zn] (SOD3) was identified as an upregulation in our study, suggesting an increase in the formation of free radicals in the hepatic tissue during the worsening of hepatitis non-HepA-E, a consequence that possibly favors the progression process of liver damage as well as increased cell signaling and interference with tissue repair.

SOD3, as well as other enzymes related to the antioxidant system, is important in protecting cells and tissues by direct action against free radicals, which are formed in an exacerbated way as a result of physiological changes often caused by infections and various pathologies.^47^ Severe liver damage, as occurs in hepatitis, is capable of increasing the formation of superoxide ions and the upregulation of antioxidant enzymes, thus increasing oxidative stress.^48^

## 4 Conclusions

The study of the plasma proteome allowed us to investigate the mechanisms that possibly favor the rapid worsening of non-HepA-E hepatitis in children as well as some respiratory viruses as potential biological triggers of the disease. We demonstrated the possibility of high-resolution mass spectrometry proteomics as a promising tool in the prediction of severity level as well as therapeutic targets for the control of liver fibrosis in potential cases of non-HepA-E hepatitis and other rapidly progressive liver diseases. Further studies with a larger number of samples aiming to determine biomarker proteins, as well as those associated with viral pathogens as possible causes, show promise.

## 5 Materials and Methods

### 5.1 Experimental Design and Obtaining Clinical Samples

We performed a clinical case study approach focusing on non-HepA-E hepatitis. Two plasma samples were used: a positive sample (test sample) and a negative sample serving as a control. Both samples were obtained from patients of Instituto de Medicina Integral Professor Fernando Figueira—IMIP Hospital (Recife, PE—Brazil). A 13 month-old female child who initially tested positive for hepatitis A, B, C, D, and E was selected for the positive sample. The analysis included transaminase indices, aspartate aminotransferase (AST), and alanine aminotransferase (ALT), along with immunoserological testing for viral hepatitis. The negative sample was obtained from another female child, aged 16 months, who also underwent the same tests and was diagnosed without any hepatitis. All samples were obtained after approval by the Research Ethics Committee (CEP) of the Federal University of Pernambuco, Brazil, for studies with biological samples from human beings.

### 5.2 Sample Preparation

#### 5.2.1 Depletion and Precipitation

The albumin and immunoglobulin G (IgG) depletion of the plasma samples was carried out using the Albumin and IgG Depletion SpinTrap Kit (Cytiva, ref 28-9480-20). To quantify total proteins, the Pierce BCA Protein Assay kit (Thermo ref 23225) was used, with dilution 10/20 times, according to the manufacturer’s protocol. For the execution of the following processing procedures, a maximum concentration of 100 μg of protein for 100 μL of the sample was established. Subsequently, the process of protein precipitation and cleaning of the samples was carried out to eliminate possible nonprotein interferents using the 2-D Cean-Up Kit (Cytiva, ref 80-6484-51).

#### 5.2.2 Proteolytic Digestion

The proteolytic digestion process was carried out using trypsin as a cleavage enzyme. Initially, to break the hydrophobic interactions, the previously processed pellets were resuspended with 50 μL of 8 M urea. Then, to reduce disulfide bonds, 2,5 μL of 100 mM DTT was added, vortexed, and incubated for 30 min at 30 °C. Subsequently, 2,5 μL of 300 mM iodoacetamide was added and incubated for 30 min in the dark to carry out the alkylation of the disulfide bonds.

After, 350 μL of 50 mM ammonium bicarbonate, pH 7.8, and 10 μL of Trypsin Gold Promega (0.5 μg/μL) were added, followed by incubation in a water bath (37 °C) overnight for 18 h. Subsequently, centrifugation was performed at 11,000*g* for 10 min at 4 °C, where the supernatant was transferred to LoBind tubes. The tubes were taken to speedVac for considerable volume reduction and stored in an ultrafreezer at −80 °C until use in the following steps.

### 5.3 Proteomic Analysis

#### 5.3.1 LC–MS/MS Analysis

The analyses were carried out in high-performance liquid chromatography using the M-Class ACQUITY UPLC nanoflow system (Waters Corporation, USA) coupled to a Hybrid Quadrupole Time of Flight Mass Spectrometer (ESI-qTOF MS/MS) model SYNAPT XS (Walters) for tryptic peptides fractionation. As the stationary phase, three columns were used: the first dimension is a first 1D column (5 μm nanoEase M/Z Peptide BEH130 C18, 300 μm × 50 mm) operating at 2 μL/min. In this first stage, mobile phase A was used, containing additional water and 0.1% (v/v) formic acid, and mobile phase B was composed of acetonitrile. The peptides were then eluted and separated by 5 fractions of mobile phase B (11.4, 14.7, 17.4, 20.7, and 50%) for 70 min each, for 9.5 min charging rate.

In the second dimension, a nanoflow Trap column was used (5 μm NanoEase M/Z Symmetry C18, 180 μm × 20 mm) coupled to a third analytical type column (1.8 μm nanoEase M/Z HSS C18 T3, 75 μm × 150 mm), with an operating mode of 0.4 μL/min and a temperature of 35 °C. For the elution of the peptides, 2 flows were performed: the first with 3–45% of mobile phase B for 46 min and the second with acceleration of mobile phase B to 90% lasting 4 min and then an equilibrium at 3% of mobile phase B for 20 min.

The system was coupled to a Hybrid Quadrupole Time-of-Flight Mass Spectrometer (Q-ToF MS/MS) model SYNAPT XS (Waters Corporation, United Kingdom). Mass spectra with the mass/charge ratio (*m*/*z*) were obtained and the positive mode of operation of the mass spectrometer with a resolution of 30.000, fwhm. The ESI low flow probe was operated with the following parameters: capillary voltage of 3 kV; source offset of 30 V; source temperature of 100 °C; sampling cone of 40 V; and cone gas of 50 L/min.

Ionic fragmentation was carried out in a collision chamber with argon gas, and ion mobility was used to separate possible ions equivalent to peptides belonging to protein isoforms using helium gas for this process. The time-of-flight (ToF) mass analyzer was externally calibrated with NaCsI from *m*/*z* 50 to 2000. And a mass reference signal locking GluFibrinopeptide B (*m*/*z* 785.8426) was obtained every 30 s. Data were acquired using the UDMSE acquisition mode, and all data were acquired in triplicate.

### 5.4 Proteomic Data Analysis

Data analysis was performed using the PROGENESIS QI (Nlinear Dynamics) Waters software, version: 4.7, to identify proteins through deconvolution of the raw mass spectra obtained. All proteins present in the sample were determined using the UniProt (Universal Protein Knowledgebase) database for proteins from the species *Homo sapiens*—human (downloaded on November 26, 2022).

For initial processing, the following parameters were used: fixed modification of carbamidomethyl to cysteine (Cys), variable modification in methionine oxidation (Met), maximum protein mass value of 650 kDa, and loss of trypsin cleavage. Proteins were then identified using a search threshold of 2 minimum peptides per protein, 2 minimum fragments per peptide, 5 minimum fragments per protein, and a FDR = 1.

A total of three most abundant peptides for all identified proteins (Hi-label-free method) were used as a procedure for relative quantification. Only proteins with confidence interval values and frequency scores above 99% are considered viable for investigation in the databases. Results such as mass, peptide quantity, unique peptides, and normalized abundance values were determined. The matrix resulting from preprocessing and identification in Progenesis was imported into the Perseus software (MaxQuant) for data filtering and statistical analysis.

### 5.5 Statistical Analysis

PERSEUS software version 2.0.7.0 (MaxQuant) was used to perform statistical analyses.^49^ After importing the matrix into Perseus, a logarithmic transformation was performed in base 2 (log2(*x*)) to ensure a normal distribution of values. Subsequently, the columns with the data were filtered into valid values (in each of the sample groups), and a categorical annotation of lines was made. After these steps, a Student’s *t*-test was performed for two test samples with minimum values of Fold Charge (s0) = 1.5 and FDR = 0.01. Thus, it was possible to determine the distribution of differentially expressed DEP proteins (criteria: *p* value < 0.05 and LogFC < −0.37 or >0.37), correlation between replicates of each sample (using MultiScatter graph with correlation values Pearson), and significance level of protein expressions by volcano graph. Next, a *Z*-score was performed to create a heat map to show the distribution of DEPs between the replicates of each sample.

### 5.6 Protein–Protein Interaction and Functional Analysis

Functional analysis was performed using Cytoscape software version 3.10.2, using protein accession codes (UniProt) to generate the protein–protein interaction network. The Omics visualizer plugin was used to identify DEPs in the main interaction network by using the Log(Fold change) values of each protein. To identify hubs, we used the M code plugin, and to determine the most connected genes in the network (hub genes), we used Cytohuba.

We used Metascape version v3.5.2030101^50^ for enrichment analyses and evaluation of which biomolecular pathways each group of up- and down-regulated proteins, the possible associations of these pathways with diseases, how these proteins possibly regulate these mechanisms, as well as the signature by the gene expression of cells and tissues of specific action.
